# Polymorphisms, Mutations, and Amplification of the *EGFR* Gene in Non-Small Cell Lung Cancers

**DOI:** 10.1371/journal.pmed.0040125

**Published:** 2007-04-24

**Authors:** Masaharu Nomura, Hisayuki Shigematsu, Lin Li, Makoto Suzuki, Takao Takahashi, Pila Estess, Mark Siegelman, Ziding Feng, Harubumi Kato, Antonio Marchetti, Jerry W Shay, Margaret R Spitz, Ignacio I Wistuba, John D Minna, Adi F Gazdar

**Affiliations:** 1 Hamon Center for Therapeutic Oncology Research, University of Texas Southwestern Medical Center, Dallas, Texas, United States of America; 2 Cancer Prevention Research, Public Health Sciences, Fred Hutchinson Cancer Research Center, Seattle, Washington, United States of America; 3 Department of Pathology, University of Texas Southwestern Medical Center, Dallas, Texas, United States of America; 4 First Department of Surgery, Tokyo Medical University, Tokyo, Japan; 5 Pathology Unit, Clinical Research Center, Center of Excellence on Aging, University Foundation, Chieti, Italy; 6 Department of Cell Biology, University of Texas Southwestern Medical Center, Dallas, Texas, United States of America; 7 Department of Epidemiology, University of Texas M. D. Anderson Cancer Center, Houston, Texas, United States of America; 8 Department of Pathology, University of Texas M. D. Anderson Cancer Center, Houston, Texas, United States of America; 9 Department of Internal Medicine, University of Texas Southwestern Medical Center, Dallas, Texas, United States of America; 10 Department of Pharmacology, University of Texas Southwestern Medical Center, Dallas, Texas, United States of America; Memorial Sloan-Kettering Cancer Center, United States of America

## Abstract

**Background:**

The *epidermal growth factor receptor (EGFR)* gene is the prototype member of the type I receptor tyrosine kinase (TK) family and plays a pivotal role in cell proliferation and differentiation. There are three well described polymorphisms that are associated with increased protein production in experimental systems: a polymorphic dinucleotide repeat *(CA simple sequence repeat 1 [CA-SSR1])* in intron one (lower number of repeats) and two single nucleotide polymorphisms (SNPs) in the promoter region, −216 (G/T or T/T) and −191 (C/A or A/A). The objective of this study was to examine distributions of these three polymorphisms and their relationships to each other and to *EGFR* gene mutations and allelic imbalance (AI) in non-small cell lung cancers.

**Methods and Findings:**

We examined the frequencies of the three polymorphisms of *EGFR* in 556 resected lung cancers and corresponding non-malignant lung tissues from 336 East Asians, 213 individuals of Northern European descent, and seven of other ethnicities. We also studied the *EGFR* gene in 93 corresponding non-malignant lung tissue samples from European-descent patients from Italy and in peripheral blood mononuclear cells from 250 normal healthy US individuals enrolled in epidemiological studies including individuals of European descent, African–Americans, and Mexican–Americans. We sequenced the four exons (18–21) of the TK domain known to harbor activating mutations in tumors and examined the status of the *CA-SSR1* alleles (presence of heterozygosity, repeat number of the alleles, and relative amplification of one allele) and allele-specific amplification of mutant tumors as determined by a standardized semiautomated method of microsatellite analysis. Variant forms of SNP −216 (G/T or T/T) and SNP −191 (C/A or A/A) (associated with higher protein production in experimental systems) were less frequent in East Asians than in individuals of other ethnicities (*p <* 0.001). Both alleles of *CA-SSR1* were significantly longer in East Asians than in individuals of other ethnicities (*p <* 0.001). Expression studies using bronchial epithelial cultures demonstrated a trend towards increased mRNA expression in cultures having the variant SNP −216 G/T or T/T genotypes. Monoallelic amplification of the *CA-SSR1* locus was present in 30.6% of the informative cases and occurred more often in individuals of East Asian ethnicity. AI was present in 44.4% (95% confidence interval: 34.1%–54.7%) of mutant tumors compared with 25.9% (20.6%–31.2%) of wild-type tumors (*p =* 0.002). The shorter allele in tumors with AI in East Asian individuals was selectively amplified (shorter allele dominant) more often in mutant tumors (75.0%, 61.6%–88.4%) than in wild-type tumors (43.5%, 31.8%–55.2%, *p =* 0.003). In addition, there was a strong positive association between AI ratios of *CA-SSR1* alleles and AI of mutant alleles.

**Conclusions:**

The three polymorphisms associated with increased EGFR protein production (shorter *CA-SSR1* length and variant forms of SNPs −216 and −191) were found to be rare in East Asians as compared to other ethnicities, suggesting that the cells of East Asians may make relatively less intrinsic EGFR protein. Interestingly, especially in tumors from patients of East Asian ethnicity, *EGFR* mutations were found to favor the shorter allele of *CA-SSR1,* and selective amplification of the shorter allele of *CA-SSR1* occurred frequently in tumors harboring a mutation. These distinct molecular events targeting the same allele would both be predicted to result in greater EGFR protein production and/or activity. Our findings may help explain to some of the ethnic differences observed in mutational frequencies and responses to TK inhibitors.

## Abstract 2

Masaharu Nomura and colleagues examine the distribution of*EGFR* polymorphisms in different populations and find differences that might explain different responses to tyrosine kinase inhibitors in lung cancer patients.

## Editors' Summary

**Background.:**

Most cases of lung cancer—the leading cause of cancer deaths worldwide—are “non-small cell lung cancer” (NSCLC), which has a very low cure rate. Recently, however, “targeted” therapies have brought new hope to patients with NSCLC. Like all cancers, NSCLC occurs when cells begin to divide uncontrollably because of changes (mutations) in their genetic material. Chemotherapy drugs treat cancer by killing these rapidly dividing cells, but, because some normal tissues are sensitive to these agents, it is hard to kill the cancer completely without causing serious side effects. Targeted therapies specifically attack the changes in cancer cells that allow them to divide uncontrollably, so it might be possible to kill the cancer cells selectively without damaging normal tissues. Epidermal growth factor receptor (EGRF) was one of the first molecules for which a targeted therapy was developed. In normal cells, messenger proteins bind to EGFR and activate its “tyrosine kinase,” an enzyme that sticks phosphate groups on tyrosine (an amino acid) in other proteins. These proteins then tell the cell to divide. Alterations to this signaling system drive the uncontrolled growth of some cancers, including NSCLC.

**Why Was This Study Done?:**

Molecules that inhibit the tyrosine kinase activity of EGFR (for example, gefitinib) dramatically shrink some NSCLCs, particularly those in East Asian patients. Tumors shrunk by tyrosine kinase inhibitors (TKIs) often (but not always) have mutations in EGFR's tyrosine kinase. However, not all tumors with these mutations respond to TKIs, and other genetic changes—for example, amplification (multiple copies) of the *EGFR* gene—also affect tumor responses to TKIs. It would be useful to know which genetic changes predict these responses when planning treatments for NSCLC and to understand why the frequency of these changes varies between ethnic groups. In this study, the researchers have examined three polymorphisms—differences in DNA sequences that occur between individuals—in the *EGFR* gene in people with and without NSCLC. In addition, they have looked for associations between these polymorphisms, which are present in every cell of the body, and the *EGFR* gene mutations and allelic imbalances (genes occur in pairs but amplification or loss of one copy, or allele, often causes allelic imbalance in tumors) that occur in NSCLCs.

**What Did the Researchers Do and Find?:**

The researchers measured how often three *EGFR* polymorphisms (the length of a repeat sequence called *CA-SSR1,* and two single nucleotide variations [SNPs])—all of which probably affect how much protein is made from the *EGFR* gene—occurred in normal tissue and NSCLC tissue from East Asians and individuals of European descent. They also looked for mutations in the EGFR tyrosine kinase and allelic imbalance in the tumors, and then determined which genetic variations and alterations tended to occur together in people with the same ethnicity. Among many associations, the researchers found that shorter alleles of *CA-SSR1* and the minor forms of the two SNPs occurred less often in East Asians than in individuals of European descent. They also confirmed that *EGFR* kinase mutations were more common in NSCLCs in East Asians than in European-descent individuals. Furthermore, mutations occurred more often in tumors with allelic imbalance, and in tumors where there was allelic imbalance and an *EGFR* mutation, the mutant allele was amplified more often than the wild-type allele.

**What Do These Findings Mean?:**

The researchers use these associations between gene variants and tumor-associated alterations to propose a model to explain the ethnic differences in mutational frequencies and responses to TKIs seen in NSCLC. They suggest that because of the polymorphisms in the *EGFR* gene commonly seen in East Asians, people from this ethnic group make less EGFR protein than people from other ethnic groups. This would explain why, if a threshold level of EGFR is needed to drive cells towards malignancy, East Asians have a high frequency of amplified *EGFR* tyrosine kinase mutations in their tumors—mutation followed by amplification would be needed to activate EGFR signaling. This model, though speculative, helps to explain some clinical findings, such as the frequency of *EGFR* mutations and of TKI sensitivity in NSCLCs in East Asians. Further studies of this type in different ethnic groups and in different tumors, as well as with other genes for which targeted therapies are available, should help oncologists provide personalized cancer therapies for their patients.

**Additional Information.:**

Please access these Web sites via the online version of this summary at http://dx.doi.org/10.1371/journal.pmed.0040125.

- US National Cancer Institute information on lung cancer and on cancer treatment for patients and professionals
- MedlinePlus encyclopedia entries on NSCLC
- Cancer Research UK information for patients about all aspects of lung cancer, including treatment with TKIs
- Wikipedia pages on lung cancer, EGFR, and gefitinib (note that Wikipedia is a free online encyclopedia that anyone can edit)

## Introduction

*Epidermal growth factor receptor* (*EGFR,* also known as *ERBB1*) belongs to the *ERBB* gene family of receptor tyrosine kinases (TKs), and is a major regulator of several distinct and diverse signaling pathways [1–3]. It is frequently overexpressed in many malignancies including non-small cell lung cancer (NSCLC), and overexpression may be associated with a negative prognosis [4,5]. A recent finding that mutations of the gene in lung cancers predict, somewhat imprecisely, response to TK inhibitors (TKIs) has generated much interest [6–10]. Mutations are limited to the first four exons of the TK domain, and occur more often in individuals with adenocarcinoma histology, East Asian origin, female gender, and never smoker status. However, exceptions exist to the correlation between mutation status and response to TKIs, suggesting that other factors may play a role. Recently, *EGFR* amplification has been identified as a further factor that may predict response to therapy [11,12]. Experimental evidence indicates that polymorphisms of the gene may also regulate protein expression.

*CA simple sequence repeat 1 (CA-SSR1)* is a highly polymorphic locus containing 14–21 CA dinucleotide repeats and is located at the 5′ end of the long intron one of the *EGFR* gene, lying upstream and in close proximity to a second enhancer [13,14]. The allele size distribution of *CA-SSR1* demonstrates ethnic differences, with East Asians having longer repeats than individuals of European descent or African–Americans [15]. By interacting with the second or downstream enhancer, a lower *CA-SSR1* repeat number was found to modulate *EGFR* transcription in vivo and in vitro, and to be correlated with increased transcription and protein expression [13,14].

The relationship between *CA-SSR1* repeat length and *EGFR* overexpression has been extensively studied in breast cancers [16,17]. Localized amplification of the *CA-SSR1* repeat, usually limited to the shorter allele, occurs frequently in breast cancers, is related to *EGFR* expression, and demonstrates a field effect, indicating that it is an early event during multistage pathogenesis [18]. In head and neck cancer, patients with a lower number of *CA-SSR1* repeats (total of both alleles < 35 repeats) had a statistically significantly increased likelihood of responding to erlotinib [19].

In addition to *CA-SSR1,* two kinds of single nucleotide polymorphisms (SNPs) in the promoter region may correlate with increased promoter activity and expression of *EGFR* mRNA. One of the SNPs is located −216 bp upstream from the initiator ATG (adenine as +1), and the change of nucleoside is guanine to thymine. This is an important binding site for the transcription factor SP1 that is necessary for activation of *EGFR* promoter activity [20]. The variant forms, −216 G/T or T/T, are more frequent in individuals of European descent and African–Americans than in Asians [21]. The other SNP, −191 C/C, is located in the *EGFR* promoter region near one of four transcription regions (−214 to −200) [22]. This SNP may also be associated with increased protein expression, and the minor forms, −191 C/A or A/A are also rare among Asians [21].

For the reasons discussed above, we investigated the distribution of these SNPs in lung cancer patients and healthy individuals of various ethnicities, the length and allelic imbalance (AI) of *CA-SSR1* in lung cancer patients, and the relationship between AI of *CA-SSR1* and allele-specific amplification in lung cancer patients with mutations of the *EGFR* gene.

## Methods

Because of the multiple, complex studies performed in this report, we summarize the salient investigations and their results in Table 1.

**Table 1:**
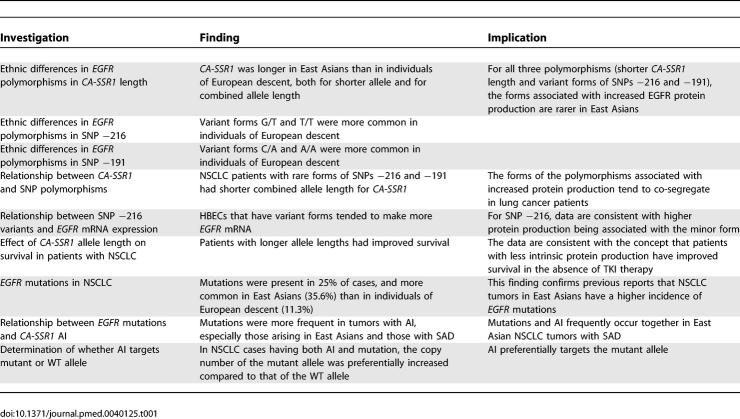
Summary of Investigations Performed, Results, and Their Implications

### Human Bronchial Epithelial Cell and Lung Cancer Cell Lines

All cancer cell lines were cultured in RPMI 1640 (Life Technologies, Rockville, Maryland, United States) supplemented with 5% fetal bovine serum and incubated in humidified air and 5% CO_2_ at 37 °C. Most cell lines were established by us at one of two locations. The prefix NCI indicates cell lines established at the National Cancer Institute, and the prefix HCC indicates cell lines established at the Hamon Center for Therapeutic Oncology Research of the University of Texas Southwestern Medical Center.

Human bronchial epithelial cells (HBECs) from healthy individuals or those with lung cancer were immortalized and cultured by us as previously described [23,24]. The cells were cultured in K-SFM medium (Life Technologies) and included 5 ng/ml EGF.

### Clinical Samples

A total of 556 samples of primary lung cancers including adenocarcinomas (*n =* 345, 62%), squamous cell carcinomas (*n =* 182, 33%), adenosquamous carcinomas (*n =* 16, 3%), and large cell carcinomas (*n =* 10, 2%) were obtained from four countries, the US, Australia, Japan, and Taiwan, and included 336 (60%) tumors from East Asians and 220 (40%) from other ethnicities (97% of whom were of European descent). None of the cases had prior treatment with TKIs. Samples of tumor containing relatively high percentages of tumor (>70%) were selected and analyzed without microdissection.

Corresponding non-malignant lung tissues were available from 450 of the samples. We also obtained 93 DNA samples from non-malignant lung tissue of European-descent patients with lung cancer in Italy and 250 DNA samples of peripheral blood mononuclear cells (PBMCs) from healthy individuals of European descent (*n =* 75), African–Americans (*n =* 75), and Mexican–Americans (*n =* 100) enrolled in ongoing epidemiological studies in the US for investigation of frequencies of the polymorphisms (Table 2). Institutional Review Board permission and informed consent were obtained at each collection site.

**Table 2:**
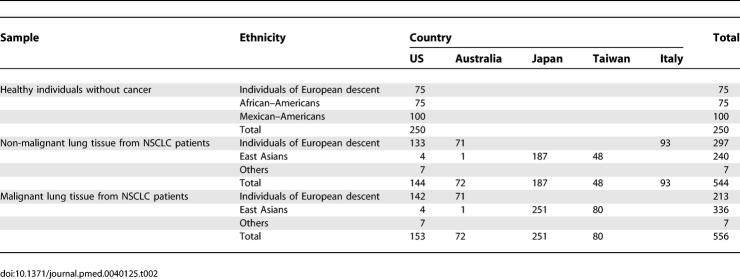
Summary of Germline (Blood) and Malignant and Non-Malignant Lung Tissues Examined

### DNA Extraction

Genomic DNA was isolated from cell lines, frozen primary tumors, and non-malignant tissues by digestion with 100 μg/ml proteinase K (Life Technologies) followed by standard phenol-chloroform (1:1) extraction and ethanol precipitation [25].

### *EGFR* Gene Mutations

Details about *EGFR* mutation types and methodologies for mutation detection have been published elsewhere [9]. Briefly, we sequenced exons 18–21 of the TK domain of *EGFR* in tumor and corresponding non-malignant tissues. The overall frequency of mutation was 20%, and there were three kinds of mutations, in-frame deletions in exon 19, missense mutations (predominantly mutation L858R in exon 21, but also in exons 18 or 20), and in-frame duplications/insertions of one to three codons in exon 20. The resistance-associated T790M mutation in exon 20 [9] was not detected in any tumor.

### Analysis of *EGFR* Polymorphic Sites

We sequenced genomic DNA encompassing the SNP sites in the promoter region of *EGFR* −216 and −191 as described previously [21], using a single PCR reaction.

The CA-repeat-containing region of intron one was amplified by PCR. The sequences of the primers were 5′-CCA ACC AAA ATA TTA AAC CTG TCT T-3′ (forward) and 5′-CTT GAA CCA GGG ACA GCA AT-3′ (reverse). For analysis of repeat allele lengths and relative ratios, instrumentation and reagents from Applied Biosystems (Foster City, California, United States) were utilized. The reverse primer was labeled with TAMRA fluorescent dye (6-FAM) at the 5′ end. The 25-μl PCR reaction mixture contained 100 ng of genomic DNA, 10× PCR buffer containing 15 mM MgCl_2_, 2 mM of each dNTP, 10 pmol of each primer, and 1.25 units of HotStart Taq DNA polymerase (Qiagen, Valencia, California, United States). After an initial denaturalization step at 95 °C for 12 min, samples were cycled 35 times as follows: 94 °C for 30 s, 60 °C for 30 s, and 72 °C for 30 s. The final extension was at 72 °C for 20 min. The size of the products (about 80 bp) was confirmed by electrophoresis on 2% agarose gels. After PCR, 1 μl of the product plus 0.5 μl of Genescan 500 ROX molecular weight standard were denatured in 12 μl of Hi-Di Formamide (Applied Biosystems) and separated with a Prism Genetic Analyzer and analyzed by Gene Scan Analysis software 3.1 (Applied Biosystems).

**Figure 1:**
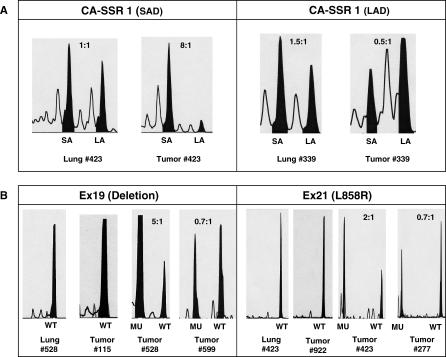
Determination of AI for Heterozygous for *CA-SSR1* and for Tumors Having a Deletion Mutation in Exon 19 or the L858R Mutation in Exon 21 Representative wave patterns are illustrated for (A) the *CA-SSR1* allele and (B) the deletion mutation in exon 19 or L858R mutation in exon 21. Both tumors and corresponding lung tissue were analyzed. Note in (A) the ratio of shorter allele to longer allele is actually 1.3:1, as illustrated for lung #423, due to artifactual preferential amplification of the short allele. Thus, an appropriate correction factor is applied.

Examination of the resultant traces demonstrated that biallelic (heterozygous) samples showed two sets of waves and two peaks, while the monoallelic (homozygous) samples showed a single set of waves and one peak (Figure 1). The highest peak reflects the repeat number of the *CA-SSR1* allele as determined by the size marker, while the preceding waves (stutter bands) represent PCR-induced artifacts. In samples without AI the shorter peak appears artificially larger as a result of preferential PCR amplification. In non-malignant lung tissue the alleles were presumed to be of equal size, and their ratios were used as a correction factor for this artificial discrepancy.

The degree of the amplification of each allele was indicated by the area under the peak as determined by software provided by the instrument's manufacturer. The relative ratios (AI ratios), termed LOH score in previous reports, of the two peaks (shorter peak area under the curve to longer peak area under the curve) in tumor samples were calculated as previously described [26]. The AI ratio was calculated thus: AI ratio = (T1 × N2)/(T2 × N1), where T indicates tumor, N indicates normal, 1 indicates the area under the peak for the shorter allele, and 2 indicates the area under the peak for the longer allele.

As either peak could be increased in relative size, AI cases were divided into shorter allele dominant (SAD) or longer allele dominant (LAD) cases. We used the definitions of these two categories as determined previously [26]. SAD cases are defined as cases in which the adjusted AI ratio was greater than 1.27, and LAD cases were those in which the adjusted AI ratio was less than 0.79. For LAD cases, the formula results in ratio values less than unity. Therefore, the ratio was inverted for LAD cases, allowing the AI ratios to reflect the relative size of the longer allele, irrespective of which allele was increased in relative size. We confirmed the previous finding that the ratios of the areas under the curve for the two alleles in constitutional DNA on repeat testing or from different individuals are relatively constant. From an analysis of constitutional DNA from over 500 healthy individuals and cancer patients, we determined that the mean ratio of the two alleles in non-malignant tissues was 1.3, resulting from artificial preferential amplification of the shorter allele (data not shown). For tumor samples lacking corresponding non-malignant tissue, the AI was determined by the formula AI ratio = T1/(T2 × 1.3).

The primers for investigation of selective amplification of the mutant or wild-type (WT) allele of exon 19 in-frame deletions and the exon 21 point mutation L858R were designed as follows: 5′-TCA CAA TTG CCA GTT AAC GTC T-3′ (forward) and 5′-CAG CAA AGC AGA AAC TCA CAT C-3′ (reverse) for exon 19, and 5′-ATG AAC TAC TTG GAG GAC CGT C-3′ (forward) and 5′-TGC CTC CTT CTG CAT GGT ATT C-3′ (reverse) for exon 21. Each forward primer was labeled with TAMRA fluorescent dye (6-FAM) at the 5′ end. The conditions for PCR were the same as for *CA-SSR1* except for the annealing temperature (57 °C for exon 19 and 61 °C for exon 21). The PCR products of exon 21 were cut by the restriction enzyme Sau96I (New England BioLabs, Ipswich, Massachusetts, United States) and analyzed. The size of each product (about 142 bp for mutant alleles of exon 19, 158 bp for the WT allele of exon 19, 100 bp for mutant the allele of exon 21, and 150 bp for the WT allele of exon 21) was also confirmed by electrophoresis in 2% agarose gels.

The ratio (mutant allele/WT allele) to define amplification of each mutant allele, exon 19 in-frame deletion or the L858R point mutation, was determined by ROC (receiver operating characteristics) curves using the definitive value of AI, 1.27 (data not shown). The definitive ratios for exon 19 and 21 were 0.82 (sensitivity 70%, specificity 68%) and 0.2 (sensitivity 90%, specificity 90%), respectively, and the combined definitive ratio was 0.47 (sensitivity 70%, specificity 61%). We used these ratios as cut-off values to determine whether the mutant allele was amplified. Because of the presence of various amounts of non-malignant cells in the tumor samples, amplifications of the WT allele could not be determined with certainty.

### Real-Time PCR for the Expression of *EGFR* mRNA

**Figure 2:**
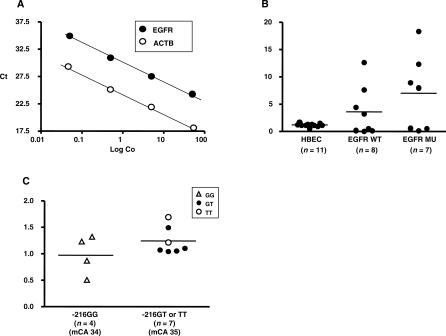
Relationship between SNP −216 Variants and *EGFR* mRNA Expression in HBEC Cultures (A) Standard curves of *EGFR* and *ACTB*. Both slopes of cycle threshold (Ct)/log copies (Log Co) were mostly coincidental. (B) Comparison of relative ratio of *EGFR/ACTB* among three groups of cultured cells (HBECs, lung cancer cell lines without *EGFR* mutations [WT], and lung cancer cell lines with *EGFR* mutations [MU]). (C) Comparison of relative ratio of HBECs having SNP −216 G/G versus G/T or T/T. mCA, mean number of *CA-SSR1* repeats.

cDNA was prepared by reverse transcription of 2 μg of RNA from cell lines using SuperScript II reverse transcriptase according to the manufacturer's protocol (Invitrogen, Carlsbad, California, United States). Real-time PCR was performed with the Sybro (SYBR) Green I method using Power SYBR Green PCR Master Mix (Applied Biosystems). *ACTB* cDNA was used as an internal control. Primer sequences were as follows: 5′-ATA GTC GCC CAA AGT TCC GTG AGT-3′ (forward) and 5′-ACC ACG TCG TCC ATG TCT TCT TCA-3′ (reverse) for *EGFR* and 5′-AGT CCT GTG GCA TCC ACG AAA CTA-3′ (forward) and 5′-ACT GTG TTG GCG TAC AGG TCT TTG-3′ (reverse) for *ACTB*. Standard curves for *EGFR* and *ACTB* were obtained (Figure 2A), and the relative expression ratios of *EGFR:ACTB* were calculated.

### Statistical Analyses

**Figure 3:**
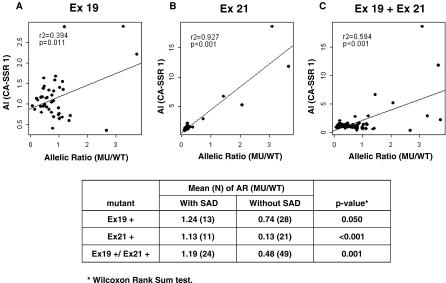
The Correlation between AI and Allelic Ratio The correlation between allelic ratio of *CA-SSR1* (shorter allele/longer allele) and the allelic ratio (AR) of mutant (MU) to WT allele of (A) the exon 19 in-frame deletion (*r* ^2^ = 0.394, *p =* 0.011), (B) the exon 21 L858R point mutation (*r* ^2^ = 0.927, *p <* 0.001), or (C) both (*r* ^2^ = 0.594, *p <* 0.001) in the same mutant cases.

We used the Chi-square test (testing the null hypothesis of equal distributions across study groups) to compare the distributions across study groups when outcomes were discrete such as genotypes of the SNP or SAD frequencies. When events were rare, e.g., where the expected cell counts were less than five, Fisher's exact test was used instead for comparisons. We also used Chi-square for an independent test for the assessment of each ethnic group using the Hardy–Weinberg equilibrium model. When outcomes were continuous, such as *CA-SSR1* repeat numbers, two-sample *t*-test and analysis of variance were used. In order to control for potential confounding bias in comparisons of SNP and *CA-SSR1* distributions, the multivariate logistic and general linear regression models were used with certain clinicopathological factors such as age, gender, smoking status, and histology as covariates (Tables 3–6). AI ratios of *CA-SSR1* plotted against mutant/WT ratios are shown in Figure 3 with the fitted regression lines. The associations between AI ratios and mutant/WT ratios were tested using Pearson's correlation for exon 19, exon 21, and both combined. To be conservative in case of small sample size and extreme values, the nonparametric Wilcoxon rank sum test was used to compare mutant/WT ratios for those with and without SAD. In this paper, all statistical tests and 95% confidence intervals are two-sided. Because of multiple tests, *p-*values less than 0.01 were judged to be statistically significant, and *p-*values less than 0.05 were judged as moderately significant. Both positive and negative results are reported in the tables and in the text.

**Table 3:**
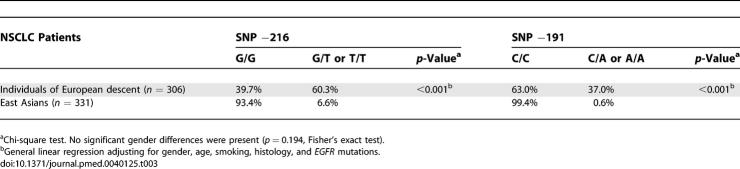
The Distribution of *EGFR* Genotypes by Ethnicity for Lung Cancer Patients

## Results

Because of the complex nature of the findings and their interrelationships, a tabular summary of our major findings is presented in Table 1.

### Ethnic Differences in Distribution of Polymorphisms

We examined ethnic differences in the distribution of the minor alleles of the two SNPs −216 and −191 in the promoter region of the *EGFR* gene and mean *CA-SSR1* repeat numbers. A summary of the samples studied from healthy individuals and cancer patients is presented in Table 2. For healthy US individuals, the frequencies of the −216 genotypes showed a borderline statistically significant difference between individuals of European descent, African–Americans, and Mexican–Americans (*p =* 0.08) (Dataset S1). The G/G genotype was present in 46.7% (95% confidence interval: 35.4%–58.0%) of individuals of European descent compared to 60% (48.9%–71.1%) and 63% (53.5%–72.5%) of African–Americans and Mexican–Americans, respectively. The frequencies of the minor forms of the −191 polymorphism were significantly lower (*p <* 0.001) in African–Americans (10.7%, 3.7%–17.7%) than in individuals of European descent (36%, 25.1%–46.9%) and Mexican–Americans (43%, 33.3%–52.7%). Also, the mean *CA-SSR1* repeat number was significantly shorter in individuals of European descent (for the shorter, longer, or combined allele lengths) than in African–Americans and Mexican–Americans (combined allele length for individuals of European descent, 35.3, 34.7–35.9, for African–Americans, 36.2, 35.6–36.8, and for Mexican–Americans, 36.8, 36.3–37.3; *p =* 0.001). The differences between African–Americans and Mexican–Americans were relatively modest and only reached significance for the shorter allele length (Dataset S1).

Among US European-descent individuals in this study, there were no significant differences in the frequency of the three polymorphisms between the healthy individuals (DNA from PBMCs) and those with NSCLC (DNA from non-malignant tissue). As shown in Table 3 and Dataset S1, the −216 G/G form was present in 46.7% (35.4%–58.0%) of the healthy individuals and 39.7% (30.8%–47.4%) of the patients with lung cancer (*p =* 0.321), and the −191 C/C genotype was present in 64% (53.1%–74.9%) of the healthy individuals and 63% (54.8%–71.2%) of the patients with cancer (*p =* 0.941). Also, the mean *CA-SSR1* repeat numbers for the short allele, long allele, and combined alleles of healthy European-descent individuals were not significantly different from those of European-descent patients with cancer (*p =* 0.492, 0.604, and 0.495, respectively) (Table 4; Dataset S1). These data permitted us to presume that the polymorphism frequencies in patients with lung cancer follow the pattern of the general population, and we can combine the data from healthy individuals and patients with NSCLC for individuals of European descent, which is the dominant ethnicity of the US, Italy, and Australia populations in this study. Furthermore, no significant differences were observed in this study for the frequencies of all three polymorphisms between individuals of European descent in the US versus in Italy, nor between East Asians in Japan versus in Taiwan (data not shown). Thus, we pooled the data from these two groups and labeled them as “individuals of European descent” and “East Asians,” which were then used for further analyses.

**Table 4:**
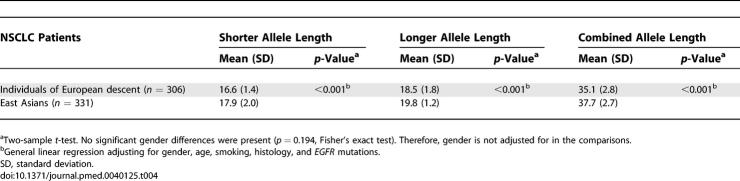
Ethnic Differences in Distribution of the Allele Lengths of *CA-SSR1* in Lung Cancer Patients

Comparing individuals of European descent and East Asians, the frequency of the minor forms of the −216 polymorphism was significantly higher (*p <* 0.001) in individuals of European descent (60.3%, 54.8%–65.8%) than in East Asians (6.6%, 3.9%–9.3%). This was also true for the minor forms of the −191 polymorphism (individuals of European descent, 37.0%, 31.6%–42.4%; East Asians, 0.6%, 0%–1.4%; *p <* 0.001), as shown in Table 3. In addition, Table 4 shows that both alleles of *CA-SSR1* (and the combined allele length) were significantly shorter in individuals of European descent than in East Asians (*p <* 0.001). The comparisons were controlled for potential confounders such as gender, age, and smoking.

### Relationship between *CA-SSR1* Allele Lengths and SNPs

We first examined the concordance of the SNP −216, SNP −191, and *CA-SSR* repeat polymorphisms. As shown in Table 5, individuals who were homo- or heterozygous for the variant forms of SNP −216 (G/T or T/T) had significantly lower mean *CA-SSR* repeat numbers in short, long, and combined allele lengths than those who were homozygous for the common form −216 G/G after adjustment for ethnicity. In similar comparisons for the variant forms of SNP −191, there was significant concordance with the longer and combined allele lengths, but not for the shorter allele.

**Table 5:**
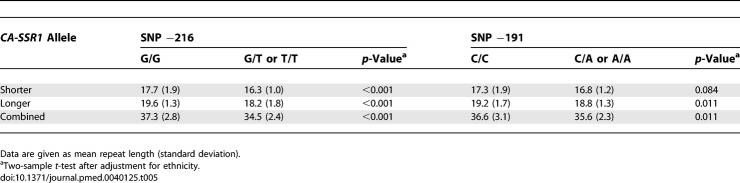
The Relationship between Repeat Length of *CA-SSR1* and SNPs

**Table 6:**
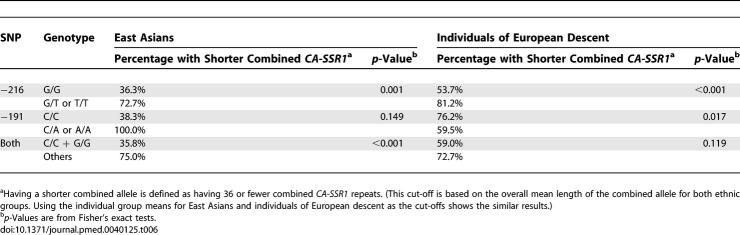
Ethnic Differences in the Relationship between the Length of *CA-SSR1* and SNPs −191 and −216

We next investigated the relationship between the combined allele length and the SNPs for different ethnicities. For convenience, since the overall mean *CA-SSR1* repeat number for shorter and longer allele combined was 36, we dichotomized the combined allele length as “longer” for those with greater than 36 repeats and as “shorter” for those with 36 repeats or fewer. As shown in Table 6, the frequency of the “shorter” combined allele was significantly higher in individuals with the minor forms of −216 (East Asians, 72.7%, 54.1%–91.3%; individuals of European descent, 81.2%, 76.1%–86.3%) than in those with the common form (East Asians, 36.6%, 30.9%–41.7%; individuals of European descent, 53.7%, 45.8%–61.6%). A similar pattern for SNP −191 was noted in East Asians but not in individuals of European descent. Also, for individuals carrying both variant genotypes of the two SNPs, the frequency of the “shorter” combined allele was observed to be higher than in those with the common forms of the SNPs in both individuals of European descent and East Asians, although the difference was statistically significant only in East Asians (Dataset S2).

### Relationship between *EGFR* Expression and the −216 Polymorphism

The polymorphism genotype of the 11 HBEC cultures was determined as previously described. The lines, derived from American individuals of European descent, showed little variation in the repeat length of the shorter *CA-SSR1* allele (mean length 16.2, range 16–17). Similarly, for the −191 polymorphism, ten of the cases had the common C/C genotype and only one case demonstrated the C/A genotype. Thus, we were unable to study the effects of these two polymorphisms on gene expression in the HBEC cultures. However, for the −216 polymorphism, four of the cases had the common form, G/G, while the remaining seven cases expressed the variant forms G/T (*n* = 5) or T/T (*n* = 2). Thus, we limited our examination of the relationship of SNPs to *EGFR* expression to the −216 polymorphism (Figure 2B and 2C).

The standard curves for *ACTB* and *EGFR* mRNA expression were straight lines nearly parallel to each other (Figure 2A), permitting us to use the expression ratio of these two genes for comparisons. To further validate our assays, we determined the ratios for the HBECs as well as for eight NSCLC cell lines having the WT form and for seven cell lines having a mutant form of the *EGFR* gene. As expression in normal epithelial cells is low or not detectable in the absence of ligand, the HBECs were cultured in EGF-containing medium (5 ng/ml). Expression in the HBECs was relatively low, with a narrow range (Figure 2B). The lung cancer lines, grown in the absence of added ligand, showed considerable variability of expression. Four WT lines had low expression, while four lines, all having *EGFR* copy number of four or greater, had considerably higher expression levels. Four of the mutant lines, all highly amplified for copy number and lacking the secondary resistance-associated T790M mutation [27,28], had high expression ratios. However three mutant lines had low expression ratios. Two of these lines had the secondary T790M mutation as well as an activating mutation, while the third line had a relatively low copy number.

While the range of expression in the HBECs was modest, we correlated expression with the −216 genotype (Figure 2C). The four lines having the G/G phenotype had a mean expression ratio of 1.0 (range 0.5–1.3). The seven lines having one of the two variant forms had a mean expression ratio of 1.2 (range 1.0–1.7). The two lines homozygous for the variant form T/T were among the three highest expressing lines. While these differences were not significant, they may represent a trend towards higher expression being associated with the variant forms.

The range of relative expression of *EGFR* compared to *ACTB* of lung cancer cell lines was variable. The two high values were observed in the cell lines with *EGFR* mutation. The mean value of cell lines having the common SNP −216 G/G (*n =* 4) was 0.97, compared to 1.24 for the lines with the minor forms SNP −216 G/T or T/T (*n =* 7) (Figure 2C). The range of the number of *CA-SSR1* repeats in the cell lines, all from individuals of European descent, was from 16 to 17 for the shorter allele, 16 to 19 for the longer allele, and 32 to 38 for the combined length. The highest value was observed in the group with the shortest combined number of *CA-SSR1* repeats (32) and one of the minor SNP −216 forms.

### The Relationship between Polymorphisms and Survival

We also investigated the relationship between the SNP −216, SNP −191, and *CA-SSR* repeat polymorphisms and patient overall survival (Figure S1). We did not observe a relationship between survival and either SNP form or any combination of SNP forms after adjusting for age, gender, ethnicity, smoking, and histology. For the shorter allele of *CA-SSR1* in the tumor cases, the mean length was 17.5. We divided the cases into those having shorter alleles, with mean lengths of 17 or fewer repeats, and those having a mean length of 18 or more repeats. We found that cases having a mean length of 18 or more repeats had improved survival compared to those having shorter allele lengths of 17 or fewer repeats (*p =* 0.017). These findings suggest that patients (in the absence of TKI therapy) whose tumor cells are predicted to make less *EGFR* protein have an improved survival compared to those whose cells are predicted to have higher intrinsic protein production. Similar data have been reported recently from another group [29]. For cases with AI of *CA-SSR1* (see below) or of the mutant allele, no differences in patient survival were noted (data not shown).

### AI of the *CA-SSR1* Alleles

The degree of amplification of each allele was reflected by the relative area under the peak (Figure 1), and the AI was determined by the ratio of shorter to longer *CA-SSR1* alleles in informative cases where two alleles were of different length. Among 450 tumor cases where the corresponding non-malignant lung tissues were available, there was no difference in the presence of homo- or heterozygosity of allele length or in the repeat length of each allele between tumor and non-malignant tissues (data not shown). These findings permitted us to analyze all 556 cases using the tumor tissues alone. For the *CA-SSR1* alleles, 376 (68%) of 556 cases were informative. The informative rate was similar to that in other previous studies [16,26]. However, in our study the informative rate was not consistent across ethnicities: there was an informative rate of 62.8 % (211/336) in East Asians and 75.0% (165/220) in other ethnicities. Of the 376 informative cases, we excluded cases with mutations other than deletions in exon 19 or the L858R mutation in exon 21 (*n* = 12) as well as patients of ethnicities other than East Asians and individuals of European descent (*n* = 5) and Asians in the US (*n =* 3). Of the remaining 356 NSCLC cases of East Asian or European descent, 263 had the WT *EGFR* gene and 95 had the mutations in exon 19 or exon 21.

For these 356 cases, we determined the ratios of the *CA-SSR1* alleles as previously described in the Methods section. AI, defined by an allelic ratio greater than 1.27 or less than 0.79, was present in 109 (30.6 %) of the cases but was significantly more frequent (*p =* 0.002) in cases with mutant tumors (44.4%, 34.1%–54.7%) than in those with WT tumors (25.9%, 20.6%–31.2%), and in East Asians (35.6%, 29.0%–42.2%) than in individuals of European descent (23.8%, 17.0%–30.6%) (*p =* 0.019) (Table 7; Dataset S3).

**Table 7:**
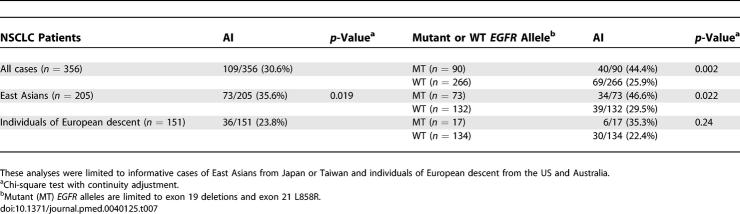
Frequencies of AI of Either Allele of *CA-SSR1* by Ethnicity

The 109 cases with AI were also divided into SAD or LAD. As shown in Table 8 (and Dataset S3), the overall frequency of SAD was 60.3% (49.1%–71.5%) in East Asians and 44.4% (28.2%–60.6%) in individuals of European descent. Also, in East Asians the SAD frequency was significantly higher (*p =* 0.001) in tumors with the exon 19 or exon 21 mutation than in those without mutations (82.4%, 69.6%–95.2%, versus 41.0%, 25.6%–56.4%). This difference, however, was not observed in patients of European descent.

**Table 8:**
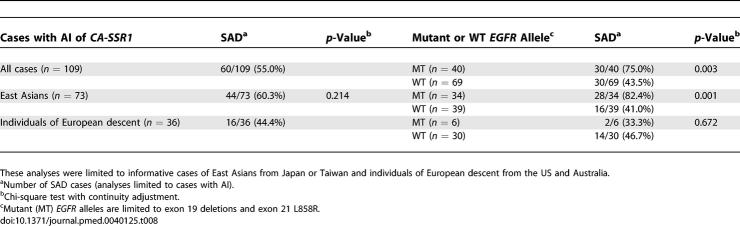
Frequencies of AI of *CA-SSR1* by Ethnicity

### AI of Mutant to WT Allele

**Figure 4:**
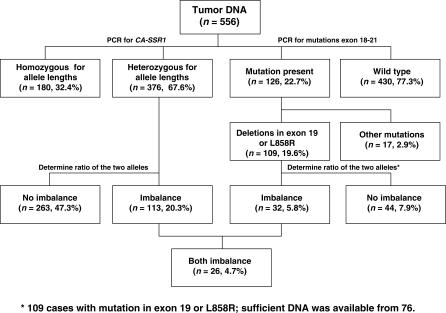
Flow Chart for Examination of the Relationship between AIs of *CA-SSR1* Length and *EGFR* Mutations

For cases with the deletions in exon 19 or the L858R mutation in exon 21, the AI of the mutant allele was determined by the mutant/WT allele ratio. A flow chart describing the process of case selection and exclusion is presented in Figure 4. These mutant cases gave us an opportunity to examine the association between AI in amplification of *CA-SSR1* repeats and AI in the ratio of mutant to WT alleles. Specifically, we wished to determine, in cases having both forms of AI, whether the mutant form was selectively amplified in association with selective amplification of the shorter allele of *CA-SSR1*. As described in the Methods section, we devised methods to determine the ratios of mutant to WT alleles for the two most frequent mutations, deletions in exon 19 and the L858R mutation in exon 21, which together account for ∼85% of *EGFR* mutations in NSCLC [9]. Of the 109 cases with mutations (in exon 19 or L858R), sufficient DNA was available from 76. Of these 76 samples, 32 (42.1%) tumors had selective imbalance involving the mutant allele. The ratio of *CA-SSR1* alleles was utilized to determine whether AI was present and, if present, which of the two alleles was preferentially overrepresented. Of these 32 samples having AI of the mutant allele, 26 (81.3%) also had AI of *CA-SSR1*. In addition, a positive correlation between AI ratios of *CA-SSR1* and mutant/WT ratios was observed in tumors having either form of mutation (Figure 3). The linear correlation was tested using Pearson's correlation and found to be significant. However, because of the possibility that the observed strong correlation might be driven by extreme values given the small sample size of the available cases, we used a nonparametric test instead to compare mutant/WT ratios between those with SAD and those without. As expected, for all the mutations under study, the cases with SAD had higher mean mutant/WT ratios than those without SAD. These findings agreed with our hypothesis that in cases demonstrating *CA-SSR1* imbalance, the mutant allele was more frequently increased in relative copy number compared to the WT allele.

## Discussion

In this report we examined the frequency of three germline polymorphisms in the *EGFR* gene in healthy individuals of different ethnicities, and in non-malignant and malignant lung tissue from patients with NSCLC. We found ethnic-related differences in polymorphism frequencies consistent with previous reports, indicating that the shorter allele of *CA-SSR1* and the minor forms of SNPs −191 (C/A or A/A) and −216 (G/T or T/T) are significantly less frequent in East Asians than in individuals of European descent [21]. In addition, we noted a relationship between the presence of the short form of *CA-SSR1* and the minor forms of the SNPs. The published data [13,19,21,26] and our observations regarding *EGFR* mRNA expression in HBECs suggest that the shorter *CA-SSR1* allele lengths and the variant forms of the −191 and −216 polymorphisms are associated with increased intrinsic gene expression. However, most of the data in the literature are from the results of transfection studies or tumor cell lines, and thus may not reflect the state of normal epithelial cells. As sections of non-malignant lung contain only a small minority of epithelial cells, a study of adjacent non-malignant lung tissues from resected cases or peripheral blood cells would not yield meaningful data. In an attempt to overcome these limitations, we studied 11 cultures of immortalized HBECs. These cultures show minimal genetic changes. In the presence of ligand stimulation, we demonstrated a trend for increased mRNA expression in lines having the SNP −216 G/T or T/T genotypes, consistent with published data. The published reports and our results are consistent with the hypothesis that cells of individuals of East Asian ethnicity express less *EGFR* protein constitutively than cells of individuals of other ethnicities. However, final experimental proof for this hypothesis is still lacking.

Amplification of the *EGFR* gene is relatively common in lung and other cancers, and may be associated with mutations of the TK domain in lung cancers [12] or of the extracellular domain in glioblastomas [30]. Two recent reports describe a correlation between copy numbers of the *EGFR* gene as measured by fluorescence in situ hybridization (FISH) and response to TKIs [11,31]. In this study we used allelic size differences in the *CA-SSR1* repeat polymorphism to determine AI of the gene. AI was observed in 30.2% of informative cases, a frequency comparable to increased copy number as detected by FISH analyses [32]. AI was significantly more frequent in East Asians and occurred nearly twice as frequently in mutant cases than in WT cases. A relationship between increased copy number by FISH analysis and mutation has also been described previously [12]. While there were no significant differences in the frequencies of either the shorter or longer allele being involved in the imbalance for all of the cases or for all of the mutant cases, in mutant cases arising in East Asians, the shorter allele was twice as likely to be preferentially amplified as the longer allele.

Finally we determined whether the mutant allele was selectively amplified in tumors having both mutation and imbalance. For tumors having deletion mutations in exon 19 or the L858R point mutation in exon 21 (together accounting for 86.5% of all mutations) we devised methods for determining the ratio of mutant to WT alleles. Of 76 cases examined, 42.1% demonstrated imbalance of the mutant allele. This figure is consistent with our finding of an overall AI (from analysis of the *CA-SSR1* alleles) percentage of 45.3% in mutant cases, and suggests that in mutation-containing tumors having AI, the mutant allele is the one that is usually amplified. Having found, by separate analyses in mutant cases, that both the shorter *CA-SSR1* allele and the mutant allele were selectively amplified, we performed a correlation of these two forms of imbalance and demonstrated a strong positive association.

**Figure 5:**
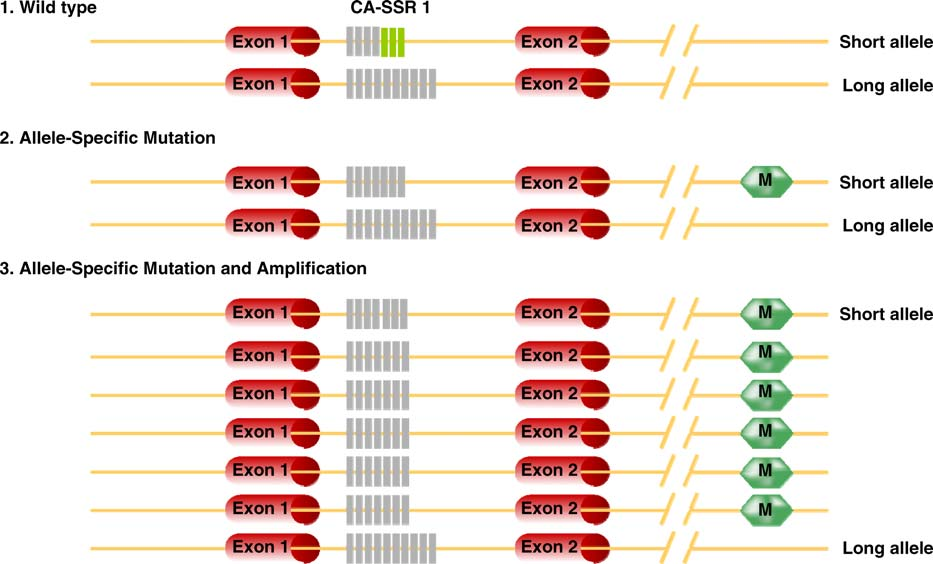
Hypothesized Allele-Specific Mutation and Amplification of *EGFR* in Lung Cancers We hypothesized that CA-SSR1 polymorphism occurs, mutations (M) target the EGFR allele with the shorter CA-SSR1 repeat number, and then there is allele-specific amplification. These three events, targeting the same allele, would be predicted to result in greater protein production than random allelic occurrence.

Incorporation of our findings and previously published data form the basis of a hypothesis suggesting a close relationship between *CA-SSR1* length, SNP −191 polymorphism, and SNP −216 polymorphism and *EGFR* gene amplification. As mentioned above, all three of these polymorphisms (shorter *CA-SSR1* length and the variant forms of the two SNPs) are reported to be associated with increased *EGFR* production, and they were rarely observed in East Asians. These findings suggest that the cells of most East Asians make less *EGFR* protein than do the cells of individuals of other ethnicities. If a certain critical level of *EGFR* is required to drive the cell toward a malignant phenotype, mutations of the TK domain and autonomous activation of downstream signaling may target East Asians, the subgroup with possibly lower intrinsic protein production. Also, we found in East Asians (but not in individuals of European descent) that mutations target the shorter *CA-SSR1* allele (suggestive of greater protein production) followed by allele-specific amplification of the mutant allele. As illustrated in Figure 5, three events target the same allele: (a) shorter *CA-SSR1* repeat length, (b) activating mutation, and (c) selective amplification of the mutant allele. These interactions favor greater protein production in mutant tumors. A similar observation was made in glioblastomas, which frequently contain a mutation or splicing variant resulting in loss of much of the extracellular domain of *EGFR*. The variant form of the allele frequently demonstrated allele-specific amplification [33]. As previously mentioned, FISH technology has been used to demonstrate that *EGFR* amplification and mutation often, but not invariably, occur together [12].

### Conclusions

The three polymorphisms associated with increased EGFR protein production (shorter *CA-SSR1* length and the variant forms of SNPs −216 and −191) were found to be rare in East Asians as compared to individuals of other ethnicities, suggesting that the cells of East Asians may make relatively less intrinsic EGFR protein. Interestingly, especially in tumors from patients of East Asian ethnicity, *EGFR* mutations were found to favor the shorter allele of *CA-SSR1,* and selective amplification of the shorter allele of *CA-SSR1* occurred frequently in tumors harboring a mutation. These distinct molecular events targeting the same allele would both be predicted to result in greater EGFR protein production and/or activity. These findings may reveal what underlies some of the ethnic differences observed in mutational frequencies and responses to TKIs.

## Supporting Information

Alternative Language Abstract S1 Translation into Japanese by Masaharu Nomura (27 KB DOC)

Alternative Language Abstract S2 Translation into French by Masaharu Nomura (31 KB DOC)

Alternative Language Abstract S3 Translation into German by Masaharu Nomura (31 KB DOC)

Alternative Language Abstract S4 Translation into Spanish by Masaharu Nomura (31 KB DOC)

Dataset S1 Ethnic Differences in Polymorphisms (37 KB DOC)

Dataset S2 Relationship between the Three Polymorphisms and *EGFR* Mutations (55 KB DOC)

Dataset S3 Mutations Target the *CA-SSR1* Allele Having the Lower Number of Repeats (48 KB DOC)

Figure S1 The Prognosis of Patients Based on the Average Length of the Shorter Allele of *CA-SSR1*
Overall survival curves for patients having a short allele of *CA-SSR1* under versus over the average length (17.5). Survival was not influenced by the minor forms of the −191 or −216 polymorphisms (data not shown). Note that none of the patients received TKI therapy.
(86 KB PPT)

## Abbreviations

AI: allelic imbalance
CA-SSR1: CA simple sequence repeat 1
*EGFR,*: *epidermal growth factor receptor*
FISH: flourescence in situ hybridization
HBEC: human bronchial epithelial cell
LAD: longer allele dominant
NSCLC: non-small cell lung cancer
PBMC: peripheral blood mononuclear cell
SAD: short allele dominant
SNP: single nucleotide polymorphism
TK: tyrosine kinase
TKI: tyrosine kinase inhibitor
WT: wild-type
