# Supplementary material for: Polymorphisms, Mutations, and Amplification of the EGFR Gene in Non-Small Cell Lung Cancers
Source: PLoS Med. 2007 Apr 24;4(4):e125. doi: 10.1371/journal.pmed.0040125 (PMC1876407; doi:10.1371/journal.pmed.0040125)
Supplement: Alternative Language Abstract S1 — (27 KB DOC) [file pmed.0040125.sd001.doc]

非小細胞肺癌におけるEGFR遺伝子の遺伝子多型、突然変異及び増幅

**要旨**

**背景**

EGFR遺伝子はI型受容体TK familyの基本的なメンバーであり、細胞の増殖、分化に大切な役割を担っている。これまでに実験系においてEGFR蛋白産生の増加に関係する３つの遺伝子多型が報告されている。１つはイントロン１に存在するCA-SSR1（少ないほどEGFRの発現が増加する）であり、残りの２つはプロモーター領域に存在するSNPである（-216 (G/T or T/T) または -191 (C/A or A/A)の場合にEGFRの発現が増加する）。この研究の目的は非小細胞肺癌において、これらの３つの遺伝子多型の分布を調べ、それらの関係やEGFR遺伝子の突然変異、対立遺伝子の不均等などとの関係を調べる事である。

**方法と結果**

我々は556例の肺癌患者の癌組織と非癌組織を調べた。人種の内訳は東アジア人種336例、白色人種213例、その他7例である。 また93例のイタリアの白色人種からの非癌組織のDNAと250例の米国健常者から採取された末梢単核球からのDNA（これらの検体は米国における白色人種、アフリカ系米国人及びメキシコ系米国人の疫学的調査のために採取されたものである。）我々は癌組織のDNAにおいてEGFR遺伝子の突然変異が存在するTK領域の４つのエクソン(18-21)をシークエンスした。 またCA-SSR1の状態（異型接合、CA数、一方の対立遺伝子の相対的増幅）、突然遺伝子を持つ対立遺伝子の特異的な増幅などを標準化されたmicrosatellite分析で調べた。 遺伝子多型の異型であるSNP -216 (G/T or T/T) 及びSNP -191 (C/A or A/A)（EGFR蛋白産生の増加に関係している）の頻度は東アジア人種では他の人種に比較して少なかった　(p<0.001)。東アジア人種ではCA-SSR1の長さは他の人種に比較して長かった (p<0.001)。気管支上皮細胞を用いたEGFR遺伝子のmRNAの発現はSNP -216 G/T または T/T を示す細胞株で増加している傾向を示した。CA-SSR1が存在する部位の対立遺伝子の相対的増加（Allelic Imbalance: AI）は異型接合例の30.6%に認められ、東アジア人種に優位であった。また突然遺伝子を持つ症例では44.4% (95% CI: 34.1% - 54.7%)であり、持たない症例では25.9% (20.6% - 31.2%)であった　(p=0.002)。東アジア人種でAIと突然変異を持つ症例では短い対立遺伝子が選択的に増幅されている症例(shorter allele dominant (SAD)) (75.0% (61.6% - 88.4%)) が突然変異を持たない症例に比較して多かった　(43.5% (31.8% - 55.2%), p=0.003)。またCA-SSR1のAIと突然変異のAIには強い正相関を認めた。

**結論**

EGFR蛋白の産生に関係している３つの遺伝子多型（より短いCA-SSR1の長さ、SNPの異型）は東アジア人種においては他の人種に比較してより少ない。この事実は東アジア人種の細胞は内因性のEGFR蛋白産生が相対的に少ない事を示唆している。興味深い事に、特に東アジア人種の肺癌患者にはEGFRの突然変異がCA-SSR1がより短い対立遺伝子に選択的に存在する事が多い。これらのはっきりとした分子生物学的な出来事が同じ対立遺伝子を標的にしているという事からEGFR蛋白質のより多くの産生或いは活性が予測できる。以上の所見はEGFRの突然変異やTK阻害剤の反応に人種間に違いがある事の基礎をなすもの或いは、関係するものかもしれない。
