# Supplementary material for: Polymorphisms, Mutations, and Amplification of the EGFR Gene in Non-Small Cell Lung Cancers
Source: PLoS Med. 2007 Apr 24;4(4):e125. doi: 10.1371/journal.pmed.0040125 (PMC1876407; doi:10.1371/journal.pmed.0040125)
Supplement: Alternative Language Abstract S2 — (31 KB DOC) [file pmed.0040125.sd002.doc]

**Polymorphisms, les mutations et l'amplification du gène EGFR dans de non-petits cancers de poumon de cellule**

**RÉSUMÉ**

**Fond**

Le gène EGFR est le membre de prototype du type I récepteur la famille de TK et joue un rôle essentiel dans la prolifération de cellule et la différentiation. Il y a trois a bien décrit polymorphisms, qui sont associés à la production de protéine augmentée dans les systèmes expérimentaux : une répétition de dinucleotide polymorphe (CA-SSR1) dans intron un (baisse le nombre de répétitions) et deux SNP polymorphisms dans la région de promoteur,-216 (G/T ou T/T) et-191 (C/A ou A/A). L'objectif de cette étude était d'examiner des distributions de ces trois polymorphisms et de leurs rapports l'un à l'autre et aux mutations de gène EGFR et au déséquilibre allelic dans de non-petits cancers de poumon (NSCLC).

**Méthodes et Conclusions**

Nous avons examiné les fréquences de trois polymorphisms dans 556 cancers de poumon resected et tissus de poumon bénins correspondants de 336 Habitants de l'Asie de l'Est, 213 Blancs (c'est-à-dire les individus et les populations de descente Européenne du Nord avec la peau blanche) et 7 d'autres ethnicities. Nous avons aussi étudié 93 tissu de poumon bénin correspondant DNAs de patients Blancs de l'Italie et de 250 sang périphérique la cellule mononucléaire (PBMC) l'ADN des sujets américains en bonne santé normaux inscrits aux études épidémiologiques en incluant des Blancs, des américains africains et des américains mexicains. Nous sequenced quatre exons (de 18-21) du domaine TK connu receler les mutations activantes dans les tumeurs et ont examiné le statut des allèles CA-SSR1 (la présence de heterozygosity, répétez le nombre des allèles et de l'amplification relative d'un allèle) et l'allèle l'amplification spécifique de tumeurs de mutant déterminées par une méthode semiautomatisée standardisée pour l'analyse microsatellite. Les formes différentes de polymorphisms de SNP-216 (G/T ou T/T) et SNP-191 (C/A ou A/A) (associé à la plus haute production de protéine dans les systèmes expérimentaux) étaient moins fréquentes dans les Habitants de l'Asie de l'Est par rapport à d'autre ethnicities (p <0.001). Les deux allèles de CA-SSR1 étaient de façon significative plus longs dans les Habitants de l'Asie de l'Est par rapport à d'autre ethnicities (p <0.001). Les études d'expression en utilisant des cultures épithéliales des bronches ont démontré une tendance vers l'expression mRNA augmentée dans les cultures ayant le SNP différent-216 G/T ou génotypes T/T. L'amplification de Monoallelic du lieu géométrique CA-SSR1 a été présente à 30.6 % des cas instructifs et a préféré l'Habitant de l'Asie de l'Est ethnicity. Le déséquilibre d'Allelic (AI) a été présent à 44.4 % (CI de 95 % : 34.1 % - 54.7 %) des tumeurs de mutant par rapport à 25.9 % (20.6 % - 31.2 %) des tumeurs de type sauvages (p=0.002). Dans les tumeurs de L'Asie de l'Est avec AI, l'allèle plus court dominant a été de manière sélective amplifié dans les tumeurs de mutant (75.0 % (61.6 % - 88.4 %)) comparé à ceux avec les tumeurs de type sauvages (43.5 % (31.8 % - 55.2 %), p=0.003). Aussi, il y avait une forte association positive entre les rapports AI d'allèles CA-SSR1 et d'AI d'allèles de mutant.

**Conclusions**

Trois polymorphisms ont fréquenté la production de protéine EGFR augmentée (plus brusquement la longueur de CA-SSR1, la variante forme SNPs -216 et -191) ont été trouvés pour être rares dans les Habitants de l'Asie de l'Est en comparaison d'autre ethnicities, en suggérant que les cellules d'Habitants de l'Asie de l'Est peuvent faire relativement moins de protéine EGFR intrinsèque. D'une façon intéressante, surtout dans les tumeurs des patients d'Habitant de l'Asie de l'Est ethnicity, les mutations d'EGFR ont été trouvées pour préférer l'allèle plus court de CA-SSR1 et l'amplification sélective de l'allèle plus court de CA-SSR1 s'est produite souvent dans les tumeurs recelant une mutation. Ces événements moléculaires distincts ciblant le même allèle seraient tous prédits pour s'ensuivre dans la plus grande production de protéine EGFR et-ou l'activité. Ces conclusions peuvent sous-tendre certaines des différences ethniques observées dans les fréquences mutational et les réponses à TKIs. Nos conclusions peuvent être rattachées à certaines des différences ethniques observées dans les fréquences mutational et les réponses à TKIs.
