# Supplementary material for: Polymorphisms, Mutations, and Amplification of the EGFR Gene in Non-Small Cell Lung Cancers
Source: PLoS Med. 2007 Apr 24;4(4):e125. doi: 10.1371/journal.pmed.0040125 (PMC1876407; doi:10.1371/journal.pmed.0040125)
Supplement: Alternative Language Abstract S3 — (31 KB DOC) [file pmed.0040125.sd003.doc]

**Polymorphisms, Veränderungen und Erweiterung des EGFR Gens in nichtkleinen Zellenlungenkrebsen**

**AUSZUG**

**Hintergrund**

Das EGFR Gen ist das Prototyp-Mitglied des Empfängers des Typs I TK Familie und spielt eine Angelrolle in der Zellenproliferation und Unterscheidung. Es gibt drei gut beschrieb polymorphisms, die mit der vergrößerten Protein-Produktion in experimentellen Systemen vereinigt werden: eine polymorphe Dinucleotide-Wiederholung (CA-SSR1) in intron ein (senken Zahl von Wiederholungen), und zwei SNP polymorphisms im Befürworter-Gebiet,-216 (G/T oder T/T) und-191 (C/A oder A/A). Das Ziel dieser Studie war, Vertrieb dieser drei polymorphisms und ihrer Beziehungen zu einander und zu EGFR Genveränderungen und allelic Unausgewogenheit in nichtkleinen Lungenkrebsen (NSCLC) zu untersuchen.

**Methoden und Ergebnisse**

Wir untersuchten die Frequenzen der drei polymorphisms in 556 resected Lungenkrebsen und entsprechenden nichtbösartigen Lungengeweben von 336 Ostasiaten, 213 Weiße (d. h. Personen und Bevölkerungen des Nordeuropäischen Abstiegs mit der weißen Haut) und 7 anderer ethnicities. Wir studierten auch 93 entsprechende nichtbösartige LUNGENGEWEBE-DNA von Weißen Patienten von Italien und 250 peripherischem Blut Monokernzelle (PBMC) DNA von normalen gesunden US-Themen eingeschrieben in epidemiologische Studien einschließlich Weißer, afrikanischer Amerikaner und mexikanischer Amerikaner. Wir sequenced die vier exons (18-21) des TK Gebiets, das bekannt ist, Aktivieren-Veränderungen in Geschwülsten zu beherbergen, und untersuchten den Status der CA-SSR1 Allele (Anwesenheit von heterozygosity, wiederholen Sie Zahl der Allele, und Verhältniserweiterung eines Allels), und Allel spezifische Erweiterung von durch eine standardisierte halbautomatisierte Methode der Mikrosatellitenanalyse bestimmten Mutant-Geschwülsten. Verschiedene Formen von polymorphisms von SNP-216 (G/T oder T/T) und SNP-191 (C/A oder A/A) (vereinigt mit der höheren Protein-Produktion in experimentellen Systemen) waren in Ostasiaten verglichen mit anderem ethnicities (p <0.001) weniger häufig. Beide Allele von CA-SSR1 waren in Ostasiaten verglichen mit anderem ethnicities (p <0.001) bedeutsam länger. Ausdruck-Studien, die epithelische Bronchialkulturen verwenden, demonstrierten eine Tendenz zum vergrößerten mRNA Ausdruck in Kulturen, die den verschiedenen SNP-216 G/T oder T/T Genotypen haben. Die Monoallelic-Erweiterung des CA-SSR1 geometrischen Orts war in 30.6 % der informativen Fälle anwesend und bevorzugte Ostasiatische Ethnizität. Allelic-Unausgewogenheit (AI) war in 44.4 % anwesend (CI von 95 %: 34.1 % - 54.7 %) Mutant-Geschwülste verglichen mit 25.9 % (20.6 % - 31.2 %) wilder Typ-Geschwülste (p=0.002). In Ostasiatischen Geschwülsten mit AI wurde das kürzere Allel dominierend in Mutant-Geschwülsten (75.0 % auswählend verstärkt (61.6 % - 88.4 %)) verglichen mit denjenigen mit wilden Typ-Geschwülsten (43.5 % (31.8 % - 55.2 %), p=0.003). Außerdem gab es eine starke positive Vereinigung zwischen AI Verhältnissen von CA-SSR1 Allelen und AI von Mutant-Allelen.

**Beschlüsse**

Die drei polymorphisms verkehrten mit der vergrößerten EGFR Protein-Produktion (kürzer CA-SSR1 Länge, Variante bildet SNPs -216, und -191) wurden gefunden, um in Ostasiaten verglichen mit anderem ethnicities selten zu sein, vorschlagend, dass die Zellen von Ostasiaten relativ weniger inneres EGFR Protein machen können. Interessanterweise, besonders in Geschwülsten von Patienten der Ostasiatischen Ethnizität, wurden EGFR Veränderungen gefunden, um das kürzere Allel von CA-SSR1 zu bevorzugen, und die auswählende Erweiterung des kürzeren Allels von CA-SSR1 kam oft in Geschwülsten vor, die eine Veränderung beherbergen. Diese verschiedenen molekularen Ereignisse, die dasselbe Allel ins Visier nehmen, würden alle vorausgesagt, um auf größere EGFR Protein-Produktion und/oder Tätigkeit hinauszulaufen. Diese Ergebnisse können einigen der ethnischen Unterschiede beobachtet in mutational Frequenzen und Antworten auf TKIs unterliegen. Unsere Ergebnisse können mit einigen der ethnischen Unterschiede beobachtet in mutational Frequenzen und Antworten auf TKIs verbunden sein.
