# Supplementary material for: Polymorphisms, Mutations, and Amplification of the EGFR Gene in Non-Small Cell Lung Cancers
Source: PLoS Med. 2007 Apr 24;4(4):e125. doi: 10.1371/journal.pmed.0040125 (PMC1876407; doi:10.1371/journal.pmed.0040125)
Supplement: Alternative Language Abstract S4 — (31 KB DOC) [file pmed.0040125.sd004.doc]

**Polimorfismo, mutaciones y amplificación de el EGFR gene en no pequeños cánceres de pulmón de célula**

**EXTRACTO**

**Fondo**

El gene EGFR es el miembro de prototipo del tipo I receptor familia de TK y desempeña un papel fundamental en proliferación de célula y diferenciación. Hay tres polimorfismos bien descritos, que tienen que ver con la producción de proteína aumentada en sistemas experimentales: una repetición de dinucleotide polimorfa (CA-SSR1) en intron un (bajan el número de repeticiones) y dos polimorfismos SNP en la región de promotor, -216 (G/T o T/T) y -191 (C/A o A/A). El objetivo de este estudio era examinar distribuciones de estos tres polimorfismos y sus relaciones el uno al otro y a mutaciones de genes EGFR y desequilibrio allelic en no pequeños cánceres pulmonares (NSCLC).

**Métodos y Conclusiones**

Examinamos las frecuencias de los tres polimorfismos en 556 cánceres pulmonares resected y tejidos pulmonares no malignos correspondientes de 336 Habitantes de Asia del Este, 213 Blancos (es decir individuos y poblaciones de pendiente de Europa Norteña con la piel blanca) y 7 de otras pertenencias étnicas. También estudiamos 93 ADNs de tejido pulmonares no malignos correspondientes de pacientes Blancos de Italia y 250 sangre periférica célula mononuclear (PBMC) ADN de sujetos estadounidenses sanos normales matriculados en estudios epidemiológicos incluso Blancos, americanos africanos y americanos mejicanos. Nosotros ordenado cuatro exons (de 18-21) de la esfera TK conocida abrigar mutaciones de activación en tumores y examinaron el estado de la CA-SSR1 alleles (presencia de heterozygosity, repita el número del alleles, y amplificación relativa de un allele) y la amplificación específica allele de tumores de mutante determinados por un método semiautomatizado estandarizado del análisis microsatélite. Las formas de variante de polimorfismos de SNP -216 (G/T o T/T) y SNP -191 (C/A o A/A) (asociado con la producción de proteína más alta en sistemas experimentales) eran menos frecuentes en Habitantes de Asia del Este comparado con otras pertenencias étnicas (p <0.001). Ambos alleles de la CA-SSR1 eran considerablemente más largos en Habitantes de Asia del Este comparado con otras pertenencias étnicas (p <0.001). Los estudios de expresión usando culturas epiteliales bronquiales demostraron una tendencia hacia la expresión mRNA aumentada en culturas que tienen la variante SNP-216 G/T o genotipos T/T. La amplificación de Monoallelic del lugar geométrico de CA-SSR1 estaba presente en el 30.6 % de los casos informativos y favoreció la pertenencia étnica de Asia del Este. El desequilibrio de Allelic (AI) estaba presente en el 44.4 % (CI del 95 %: el 34.1 % - el 54.7 %) de tumores de mutante comparado con el 25.9 % (el 20.6 % - el 31.2 %) de tumores de tipo salvajes (p=0.002). En tumores de Asia del Este con AI, más corto allele dominante fue selectivamente amplificado en tumores de mutante (el 75.0 % (el 61.6 % - el 88.4 %)) comparado a aquellos con tumores de tipo salvajes (el 43.5 % (el 31.8 % - el 55.2 %), p=0.003). También, había una asociación positiva fuerte entre proporciones AI de la CA-SSR1 alleles y AI del mutante alleles.

**Conclusiones**

Los tres polimorfismos se asociaron con la producción de proteína EGFR aumentada (longitud de CA-SSR1 más corta, la variante forma SNPs -216 y -191) fueron encontrados para ser raros en Habitantes de Asia del Este comparando con otras pertenencias étnicas, sugiriendo que las células de Habitantes de Asia del Este puedan hacer relativamente menos proteína EGFR intrínseca. De manera interesante, sobre todo en tumores de pacientes de la pertenencia étnica de Asia del Este, las mutaciones de EGFR fueron encontradas para favorecer el más corto allele de la CA-SSR1 y la amplificación selectiva de más corto allele de la CA-SSR1 ocurrió con frecuencia en tumores que abrigan una mutación. Estos acontecimientos moleculares distintos que apuntan mismo allele serían todos predichos para causar la mayor producción de proteína EGFR y/o la actividad. Estas conclusiones pueden ser la base de algunas diferencias étnicas observadas en frecuencias mutational y respuestas a TKIs. Nuestras conclusiones pueden estar relacionadas con algunas diferencias étnicas observadas en frecuencias mutational y respuestas a TKIs.
