# Supplementary material for: Polymorphisms, Mutations, and Amplification of the EGFR Gene in Non-Small Cell Lung Cancers
Source: PLoS Med. 2007 Apr 24;4(4):e125. doi: 10.1371/journal.pmed.0040125 (PMC1876407; doi:10.1371/journal.pmed.0040125)
Supplement: Dataset S1 — (37 KB DOC) [file pmed.0040125.sd005.doc]

### Ethnic differences in polymorphisms

Table S1. The distribution of EGFR genotypes by ethnicity for healthy subjects as control

| Groups | SNP -216 | | | SNP -191 | | |
| --- | --- | --- | --- | --- | --- | --- |
|  | G/G | G/T+T/T | p-value** | C/C | C/A+A/A | p-value** |
| Healthy subjects*  Whites (n=75)  African Americans (n=75)  Mexican Americans (n=100) | 46.7%  60.0%  63.0% | 53.3%  40.0%  37.0% | 0.082 | 64.0%  89.3%  57.0% | 36.0%  10.7%  43.0% | <0.001 |

* Source of the DNA from healthy controls was PBMC (peripheral blood mononuclear cells).

No significant gender differences were present (p=0.194, Fisher’s exact).

** Chi-square test

Note: For -191C/A, Whites/Mexican Americans vs. African Americans, p<0.001; Whites vs. Mexican Americans, p=0.249.

Table S2. Ethnic differences in distribution of the allele length of CA-SSR1 in healthy subjects

|  | Shorter Allele length  Mean (SD) | | Longer Allele length  Mean (SD) | | Combined Allele length  Mean (SD) | |
| --- | --- | --- | --- | --- | --- | --- |
| Healthy subjects  Whites (n=75)  African Americans (n=75)  Mexican Americans (n=100) | 16.6(1.3)  16.9(1.4)  17.4(1.6) | p-value*  0.001 | 18.7(1.8)  19.3(1.9)  19.4(1.4) | p-value*  0.015 | 35.3(2.6)  36.2(2.8)  36.8(2.5) | p-value*  0.001 |

* ANOVA test

Note: The significance at Shorter Allele arises from the difference between Mexican Americans vs. African Americans/Whites; the significance at longer allele and combined allele arise from the differences between Whites vs. African Americans/Mexican Americans.

No significant gender differences were present (p=0.194, Fisher’s exact). Therefore, gender is not adjusted for the above comparisons.
