# Supplementary material for: Polymorphisms, Mutations, and Amplification of the EGFR Gene in Non-Small Cell Lung Cancers
Source: PLoS Med. 2007 Apr 24;4(4):e125. doi: 10.1371/journal.pmed.0040125 (PMC1876407; doi:10.1371/journal.pmed.0040125)
Supplement: Dataset S2 — (55 KB DOC) [file pmed.0040125.sd006.doc]

Relationship between the three polymorphisms and *EGFR* mutations

In this study we considered three polymorphisms, SNP -191 C/A, SNP -216 G/T, and CA-SSR1 repeats. We have shown the distributions of these polymorphisms among different ethnicities (Tables S1, S2 in Dataset S1 and Text tables 3, 4). Now we would like to explore more about their association with *EGFR* mutations in lung cancer patients. The *EGFR* mutations were almost equally distributed among different polymorphisms in East Asians (Table S3). For example, for SNP -191 and -216 combined which is categorized as having common type in both -191 and -216 (C/C and G/G) versus having any rare type in either -191 or -216 (‘Others’), 31.5% of those with C/C and G/G had *EGFR* mutations while the percentage was 34.8% for ‘Others’ (p=0.817). However, in Whites we observed that the *EGFR* mutations were more likely to associate with the common G/G in SNP -216, with the C/C and G/G in SNP -191 and -216 combined, and with the longer combined CA-SSR1 allele, although the tests did not show any significance due to the low occurrence of the mutations in Whites. For example, for SNP -191 and -216 combined, 15.4% had the mutations among C/C and G/G, but only 7.3% among ‘Others’ (p=0.12).

Table S3. Relationship between polymorphisms and *EGFR* mutations (Exon19 and/or Exon21) by ethnicity.

|  | East Asians | | Whites | |
| --- | --- | --- | --- | --- |
|  | % mutation | p-value*** | % mutation | p-value*** |
| SNP-191 |  |  |  |  |
| C/C | 31.6% | 0.534 | 7.7% | 0.453 |
| C/A or A/A | 50.0% |  | 10.8% |  |
| SNP-216 |  |  |  |  |
| G/G | 31.6% | 1.0 | 13.4% | 0.078 |
| G/T or T/T | 33.3% |  | 5.7% |  |
| -191+-216 * |  |  |  |  |
| C/C and G/G | 31.5% | 0.817 | 15.4% | 0.120 |
| Others | 34.8% |  | 7.3% |  |
| CA repeats combined** |  |  |  |  |
| Longer | 31.2% | 0.808 | 11.5% | 0.422 |
| Shorter | 32.5% |  | 7.7% |  |

Since we observed that the CA-SSR1 polymorphisms also strongly associated with SNP -216 G/T in both East Asians and Whites, and with SNP -191 and -216 combined in East Asians, we further examined the association between the SNPs and the *EGFR* mutations but with stratification by CA-SSR1 repeats in case the association were confounded or modified by CA-SSR1 repeats. (Text table 6)

After the stratification, the similar association was observed. In particular, in East Asians the percentages of *EGFR* mutations between C/C and G/G in SNP -191 and -216 combined and ‘Others’ were very close among both with longer and with shorter CA-SSR1 repeats. However in Whites, the percentage of *EGFR* mutations was higher in C/C and G/G than ‘Others’ among both with longer and with shorter CA-SSR1 repeats, although the tests did not show any significance due to the low occurrence of the mutations in Whites. The results are shown in Table S4.

Table S4. Percentage of *EGFR* mutations by ethnicity by CA-SSR1 repeats.

|  |  |  | SNP -191+ -216 * | |
| --- | --- | --- | --- | --- |
|  | CA-SSR1 | *EGFR* mutations | C/C and G/G | Others |
| East Asians | Longer | % mutation | 31.1% | 33.3% |
|  |  | p-value*** | 1.0 | |
|  | Shorter ** | % mutation | 32.1% | 35.3% |
|  |  | p-value*** | 0.787 | |
| Whites | Longer | % mutation | 18.8% | 8.9% |
|  |  | p-value*** | 0.365 | |
|  | Shorter ** | % mutation | 13.0% | 6.7% |
|  |  | p-value*** | 0.385 | |

* -191+-216 is a combination of SNP-191 and -216 which is dichotomized as -191 C/C and -216 G/G versus with any rare type.

** The shorter allele is defined by <=36 combined CA-SSR1 repeats. (Using different mean as cutoff for East Asian and Whites separately shows the similar results)

*** p-values are from Fisher’s exact tests.
