# Supplementary material for: Polymorphisms, Mutations, and Amplification of the EGFR Gene in Non-Small Cell Lung Cancers
Source: PLoS Med. 2007 Apr 24;4(4):e125. doi: 10.1371/journal.pmed.0040125 (PMC1876407; doi:10.1371/journal.pmed.0040125)
Supplement: Dataset S3 — (48 KB DOC) [file pmed.0040125.sd007.doc]

**Mutations target the CA-SSR1 having the lower number of repeats**

Our hypothesis about *EGFR* gene mutations and polymorphic dinucleotide repeat CA-SSR 1 is that the mutations target the shorter CA-SSR 1 allele. And it was reported that there was selective amplification of the shorter allele of CA-SSR 1 in tumor cells. So it is reasonable to assume that the amplification favors the allele with mutations as well. 　The challenge to proof our hypotheses is that the amplification could not be measured directly since in this study the tumor tissues were examined and the percentages of tumor cells in the tissue may vary from about 20 to 70% and were not ascertainable. However, we did measure the ratios of shorter to longer allele among all informative cases where two alleles are of different length. The ratios greater than 1.27 (SAD) or less than 0.79 (LAD) were regarded as allelic imbalance (AI). The amplification was more common in mutant cases (Ex19 + and/or Ex21 +), where 44.4% had AI, versus 25.9% in wild type cases (p=0.002). And the difference was more significant in East Asians (46.6% vs. 29.6%, p=0.022) than in Whites (35.3% vs. 22.4%, p=0.24) (Text table. 7). And if the evidence of amplification presented, the shorter allele was more likely to be amplified in mutant cases. The percentage of SAD in mutant cases was 75% which was significantly higher than the 43.5% in wild type cases (p=0.003). Similarly, the significant was only observed in East Asians (82.4% vs. 41%, p=0.001) but not in Whites (Text table. 8). Table S5 shows the emphasized the part of Text table 7 and Table S6 shows the emphasized the part of Text table 8.

Table S5. Percentage of allelic imbalance (AI) for mutant (Exon19 or Exon21) vs. wild type:

|  | | AI+ | AI- | p-value* |
| --- | --- | --- | --- | --- |
| All Cases | Mutant** (N=90) | 44.4% | 55.6% | 0.002 |
|  | Wild type (N=266) | 25.9% | 74.1% |  |
| East. Asians | Mutant** (N=73) | 46.6% | 53.4% | 0.022 |
|  | Wild type (N=132) | 29.6% | 70.4% |  |
| Whites | Mutant** (N=17) | 35.3% | 64.7% | 0.240 |
|  | Wild type (N=134) | 22.4% | 77.6% |  |

* Chi-square with continuity adjustment. ** Exon19 mutation + or Exon21 mutation +

Table S6. Among AI patients, percentage of AI due to higher amplified SA (i.e. AI for shorter/longer > 1.27):

|  | SAD | LAD | p-value* |
| --- | --- | --- | --- |
| All Cases | 55.1% | 44.9% |  |
| Mutant** (N=40) | 75 % | 25% | 0.003 |
| Wild type (N=69) | 43.5% | 56.5% |
| East Asians | 60.3% | 39.7% |  |
| Mutant** (N=34) | 82.4% | 17.6% | 0.001 |
| Wild type (N=39) | 41.0% | 59.0% |
| Whites | 44.4% | 55.6% |  |
| Mutant** (N=6) | 33.3% | 66.7% | 0.672 |
| Wild type (N=30) | 46.7% | 53.3% |

* Chi-square with continuity adjustment. ** Exon19 mutation + or Exon21 mutation +

Besides AI, the ratios of mutant (MU: mutant) to non-mutant (WT: wild type) allele were measured for mutant cases. If our hypotheses were appropriate that the *EGFR* mutations preferentially target the shorter CA-SSR 1 allele, the amplification favors the shorter allele and therefore favors the mutated allele, we would expected to see the ratios of shorter to longer allele went up as the ratios of mutant to non-mutant allele went up. The AI (shorter/longer CA-SSR 1 allele) ratios were plotted against the allelic ratio (AR) (MU/WT) for each mutation type and are shown in Text figure 3.

In each case, a linear regression line was fitted and the estimated association between two ratios was estimated to be positive as expected and the association was significant using Pearson correlation test. However as shown in the first and third panel of the above plot, the observed positive correlations for Exon19 mutations or Exon19/Exon21 combined might be driven by a few cases with extreme values.

So instead of making any assumptions on the distribution and the linearity, we used the nonparametric Wilcoxon Rank-Sum tests to compare the average AR between those with SAD and without SAD among mutant cases. The expected positive association between the AI ratios and the AR was also observed here for each mutation or combined, where those with SAD had higher mean AR than those without, and the difference was tested to be significant. The detailed results are shown in Text figure 3.

So far we have shown that our proposed hypotheses about the association between *EGFR* mutations, polymorphic CA-RSS 1 repeats, and amplification seemed to be appropriate in this study. However, since we used tissue samples and the amplification was not directly measured, it is necessary to further verify those hypotheses.
