# Supplementary material for: Polymorphisms, Mutations, and Amplification of the EGFR Gene in Non-Small Cell Lung Cancers
Source: PLoS Med. 2007 Apr 24;4(4):e125. doi: 10.1371/journal.pmed.0040125 (PMC1876407; doi:10.1371/journal.pmed.0040125)
Supplement: Figure S1 — Overall survival curves for patients having a short allele of CA-SSR1 under versus over the average length (17.5). Survival was not influenced by the minor forms of the −191 or −216 polymorphisms (data not shown). Note that none of the patients received TKI therapy. (86 KB PPT) [file pmed.0040125.sg001.ppt]

## Slide 1
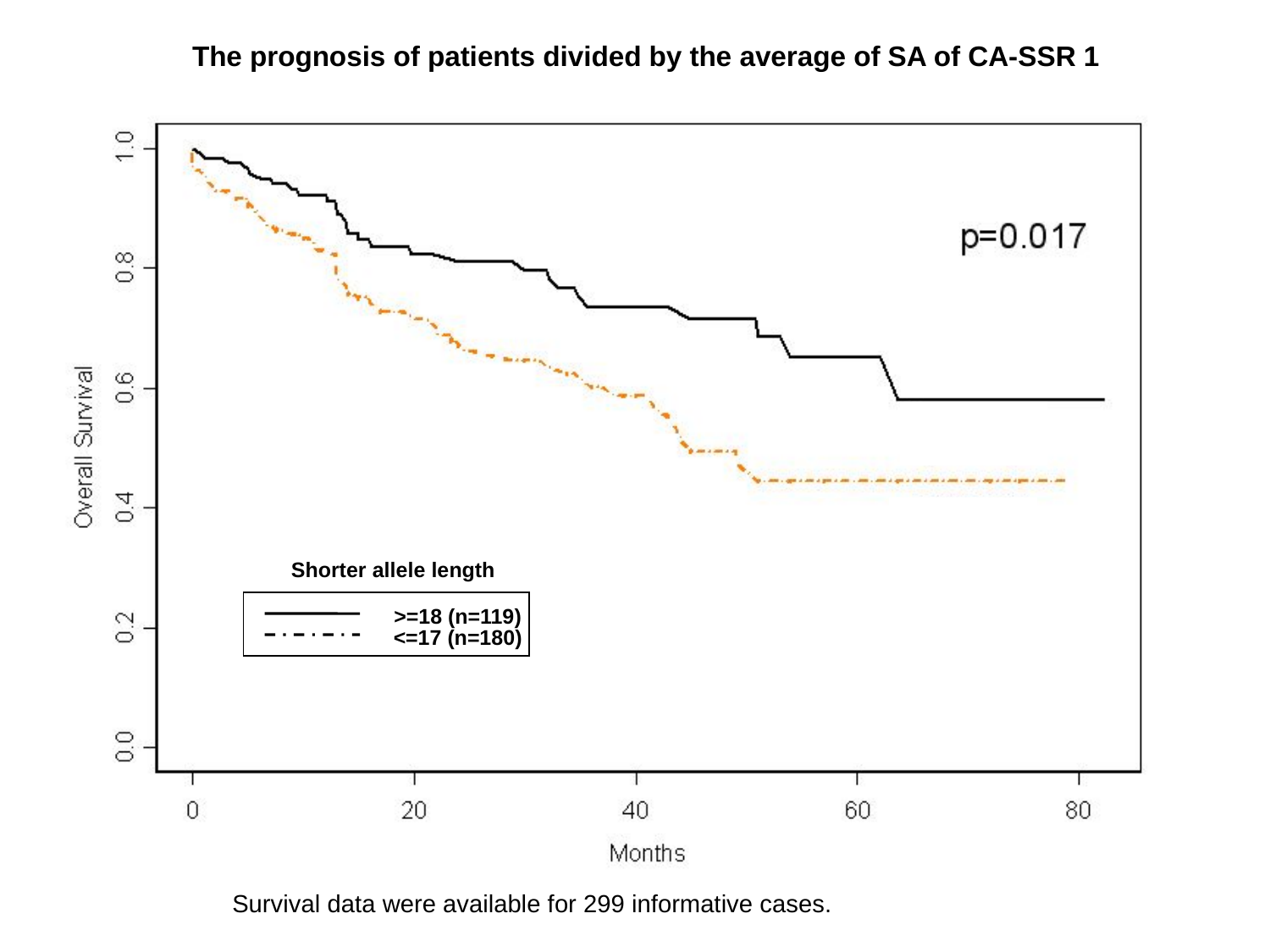

The prognosis of patients divided by the average of SA of CA-SSR 1
Shorter allele length
>=18 (n=119)
<=17 (n=180)
Survival data were available for 299 informative cases.
